# X-ray diffraction data for the C5a-peptidase mutant with modified activity and specificity

**DOI:** 10.1016/j.dib.2022.108778

**Published:** 2022-11-23

**Authors:** Todd F. Kagawa, Monica Jain, Jakki C. Cooney

**Affiliations:** a Department of Biological Sciences, University of Limerick, Limerick, Ireland; b Bernal Institute, University of Limerick, Limerick, Ireland; c SSPC, University of Limerick, Ireland

**Keywords:** C5a peptidase, Immunomodulatory enzymes, Exosite, Substrate binding, Enzyme activity, Mutant

## Abstract

The Streptococcal C5a peptidase (ScpA) specifically inactivates the human complement factor hC5a, a potent anaphylatoxin recently identified as a therapeutic target for treatment of COVID-19 infections. Engineering of ScpA to enhance its potential as a therapeutic will require detailed examination of the basis for its highly selective activity. The emerging view of ScpA and related subtilases is that selection of their substrates is a dynamic two-step process involving flexibility in the domains around the active site and in the C-ter of the substrate. Surface plasmon resonance (SPR) analyses of the ScpA-hC5a interaction have shown that high affinity binding of the substrate is driven by electrostatic interactions between an exosite on the Fn2 domain of the enzyme and the bulky N-ter cleavage product (P_N_, ’core’ residues 1-67) of C5a [1]. Introduction of a D783A mutation in the Fn2 exosite, located approximately 50 Å from the catalytic serine, was shown to significantly reduce substrate binding affinity and *k*_cat_ of the enzyme. X-ray crystallographic studies on the D783A mutant (ScpA_D783A_) were undertaken to better interpret the impact of this mutation on the specificity and activity of ScpA. Here we present the 1.9 Å X-ray diffraction data for ScpA_D783A_ and the molecular replacement solution for the structure. Both raw diffraction images and coordinates have been made available on public databases. Additional details on the related SPR and enzyme kinetics analyses on ScpA_D783A_ reported in Jain et al. [2].

**Specifications Table**

**Table:** 

Subject	Structural Biology
Specific subject area	Structure function characterization of therapeutic enzymes.
Type of data	Table Figure
How data were acquired	Single crystal X-ray diffraction data collected at the Diamond Light Source, Beamline I24
Data format	Analyzed
Parameters for data collection	Temperature: 110 K Wavelength: 0.96864 Å Exposure time: 0.126 s Detector: Pilatus3 6M
Description of data collection	Shutterless, fine slicing data collection.
Data source location	Diamond Light Source, Oxfordshire, UK
Data accessibility	Coordinates and structure factors are deposited at the Worldwide Protein Data bank (wwPDB) Repository name: wwPDB Data identification number: 7YZX Direct URL to data: https://doi.org/10.2210/pdb7YZX/pdb Raw X-ray diffraction images are deposited at X-Ray Diffraction Archive (https://xrda.pdbj.org/entry/7yzx).
Related research article	M. Jain, M. Teçza, T.F. Kagawa, J.C. Cooney, Exosite binding modulates the specificity of the immunomodulatory enzyme ScpA, a C5a inactivating bacterial protease, Comput Struct Biotechnol J. 20 (2022) 4860-4869. https://doi.org/10.1016/j.csbj.2022.08.018

## Value of the Data

• The human complement protein C5a is implicated in immunomodulatory diseases. ScpA, a C5a inactivating protease, represents a novel enzymatic approach to therapy. Introduction of a D783A mutation in the exosite for substrate binding was demonstrated to affect both the specificity and activity of the enzyme. The structure of the D783A mutant allows a more complete interpretation of enzyme kinetic and SPR data, thus an important component of the development of ScpA into a novel therapeutic.
• Understanding how substrate specificity is achieved in these enzymes provides critical knowledge for engineering enzymes with novel medical and industrial applications.
• The structure, along with biochemical data, will inform more comprehensive studies focusing on the role of the ScpA exosite in determining substrate specificity and activity.

## 1 Data Description

### 1.1 Data collection and structure refinement

**Fig. 1:**
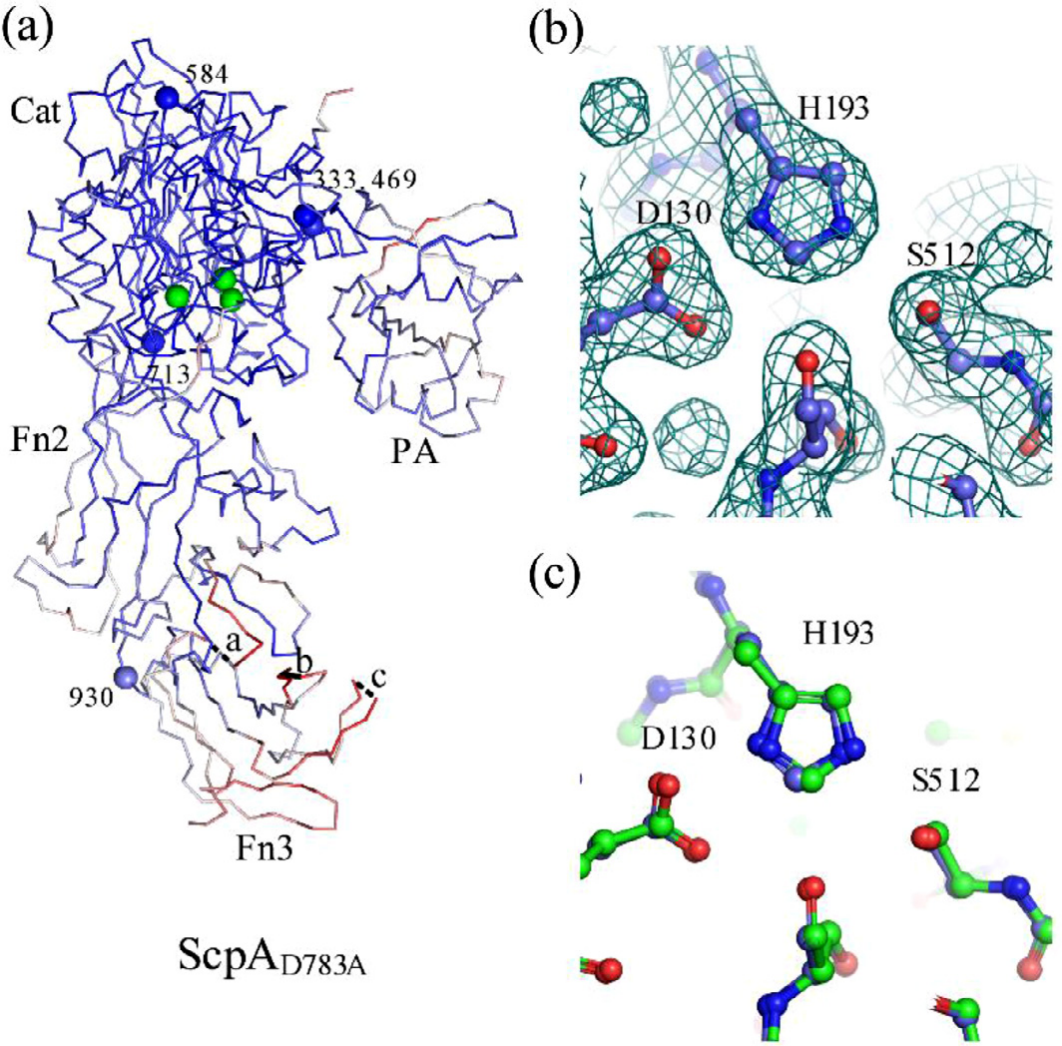
Panel a shows the Cα trace of the D783A crystal structure colored by B-factor. An RMSD of 0.457 Å was observed between the Cα of the ScpA and D783A structures. The catalytic (‘Cat’), PA, Fn2 and Fn3 domains are labelled. Cα atoms of the catalytic triad residues are shown as green spheres. Residues at the domain boundaries are drawn as spheres and labelled ‘333’, ‘469’, ‘584’, ‘713’ and ‘930’. Panel b shows the 2F_o_-F_c_ electron density map around the catalytic triad residues D130, H193 and S512 in the ScpA_D783A_. Panel c shows the superposition of the catalytic triad residues of the ScpA and D783A structures. ScpA carbon atoms are colored green. The RMSD between these residues is 0.136 Å for 24 atoms.

X-ray diffraction data to 1.9 Å resolution were collected on a single crystal of the D783A mutant of ScpA (ScpA_D783A_, referred to as ‘D783A’) at the Diamond Light Source, Beamline I24. The structure was solved by molecular replacement with PHASER [3] using the structure of ScpA as a search model (PDB code 3EIF [4]). The structure was refined with PHENIX [5] to R-work and R-free values of 19.84 and 22.89 % respectively (Fig. 1). Data collection and structure refinement parameters are shown on Table 1. The data allowed for visualization of the catalytic residues and confirmed the proper folding of the enzyme. The final model includes D783A residues 97-781, 785-972, 974-995 and 999-1030. The electron density could not be assigned to three disordered regions (residues 782-784, 973 and 996-998 labelled ‘a’, ‘b’, and ‘c’ respectively in Fig. 1a). Note that the disordered regions include the D783A mutation. Tentative assignments of electron density were made for residues 83-87 and 94-96.

**Table 1:** Data collection and refinement parameters.

*Data collection*	

Space group	P6322
Unit cell dimensions a, b, c (Å)	169.4, 169.4, 141.8
Unit cell dimensions α, β, γ (°)	90 90 120
Wavelength (Å)	0.96864
Resolution range (Å)	46.2 - 1.9
Completeness (%)	99.7 (97.9)^a^
Total reflections	1309005 (120933)
Unique reflections	93718 (9050)
Multiplicity	14.0 (13.3)
R_pim_^b^/R_meas_^c^	0.040 (1.206) /0.151 (4.639)
Mean I/sigma(I)	12.3 (0.6)
Wilson B-factor (Å^2^)	41.16
Molecules per asymmetric unit	1
*Refinement statistics*	
Reflections used in refinement	93689 (9050)
Reflections used for R-free (5% of total)	4685 (453)
R-work/R-free	0.1984 (0.3557)/0.2289 (0.3899)
CC(work)/CC(free)	0.964 (0.462)/0.951 (0.466)
Number of non-hydrogen protein atoms	7058
Number of non-hydrogen ligand/solvent atoms	48/328
Protein residues	935
Ramachandran favored, allowed, outliers (%)	96.43, 3.57, 0
RMS bonds (Å)/RMS angles (°)	0.003/0.62
Rotamer outliers (%)	0.98
Clashscore	1.74
Average B-factor (overall)	48.38
protein, ligands, solvent	48.43, 67.51, 44.44

a Numerical values in parenthesis are for highest resolution shell (1.97 - 1.90 Å).

b R_pim_=precision-indicating merging R-factor=Σ_hkl_[1/(N−1)]^1/2^ Σ_i_|I_i_(hkl)−〈I_i_(hkl)〉|/Σ_hkl_Σ_i_I_i_(hkl).

c R_meas_=redundancy-independent merging R-factor=Σ_hkl_ [N/(N−1)]^1/2^Σ_i_|I_i_(hkl)−〈I_i_(hkl)〉|/Σ_hkl_Σ_i_I_i_(hkl).

## 2 Experimental Design, Materials and Methods

### 2.1 Protein expression purification and crystallization

The cloning of recombinant ScpA D783A mutant closely followed the method described for the ScpA S512A active site mutant [1]. The pGEX-6P-3 expression vector (Cytiva, UK) with the C5a peptidase gene (coding amino-acid residues N31-S1032) from the genome of *Streptococcus pyogenes* B220 was used as a template. Residue D783 was mutated to an alanine residue with the QuikChange II site directed mutagenesis kit (Strategene, USA). The D783A mutation was confirmed by DNA sequencing. The glutathione S-transferase (GST) tagged ScpA_D783A_ mutant protein was over-expressed in *Escherichia coli* DH5α (Invitrogen, UK). Purification of D783A followed a protocol similar to that described for ScpA [1]. Briefly, the mutant protein was batch-purified from cleared cell lysates using glutathione Sepharose 4B (GE Healthcare, USA). The tag was removed by cleavage with PreScission protease, and the released protein was eluted from the affinity resin. The tag-less protein was dialyzed and further purified with ion-exchange chromatography on a Bio-Rad Econo System (Bio-Rad, USA) using a 5 mL HiTrap Q HP cartridge (Cytiva, UK) and a linear gradient of NaCl in 10 mM Tris/HCl pH 8 buffer. Peak fractions were pooled and concentrated for fractionation by size exclusion chromatography (SEC) with a Superdex 75 column (Cytiva, UK) on an Äkta Prime Plus FPLC system (Cytiva, UK) with 50 mM Hepes/KOH (pH 7.5) and 100 mM NaCl as a buffer. The peak fractions from SEC were concentrated to 11.9 mg/mL and the protein crystallization samples were aliquoted and stored at −80°C. Crystals were obtained with the hanging-drop vapor-diffusion method at 20°C, by mixing 1 μL of protein solution and 1 μL of reservoir solution [2.4 M ammonium sulfate and 0.2 M Tris/HCl (pH 7.4–7.8)]. Crystals were flash-cooled under liquid nitrogen in cryoprotectant solution [2.4 M ammonium sulfate, 0.2 M Tris/HCl (pH 7.4–7.8), and 0.8 M sodium malonate].

### 2.2 Data collection and structure solution

X-ray diffraction data were collected on single crystals at beamline I24 at the Diamond Light Source (Oxfordshire, UK). Data was collected on a Pilatus3 6M detector in shutterless mode with the wavelength set at 0.9686 Å and an exposure time of 0.126 s. The diffraction images were processed with XDS [6]. A molecular replacement solution was obtained with PHASER [3] using the X-ray crystal structure of ScpA (PDB code 3EIF [4]) as search model, with the side chains beyond Cβ atoms and regions of high B factors omitted. Residues not supported in the density maps were removed from the model prior to building. The remaining structure was built in Coot [7] and refined with PHENIX [5]. Statistics pertinent to data collection and final refinement are given in Table 1. The quality of the model was assessed with MOLPROBITY [8]. PyMOL (Schrodinger, USA) was used for visualization and rendering of figures [9]. Coordinates and structure factors have been deposited at the PDB (PDB Code 7YZX) and the raw images (https://doi.org/10.51093/xrd-00090) from data collection have been uploaded to the X-Ray Diffraction Archive (XRDa).

## Ethics Statement

All material used in this study was of bacterial origin. The authors declare compliance with the publication code of ethics of this journal.

## CRediT Author Statement

**Todd F. Kagawa:** Investigation, Conceptualization, Formal analysis, Writing – original draft, Writing – review & editing; **Monica Jain:** Investigation; **Jakki C. Cooney:** Investigation, Conceptualization, Writing – original draft, Writing – review & editing, Funding acquisition, Supervision.

## Declaration of Competing Interest

The authors declare that they have no known competing financial interests or personal relationships which have or could be perceived to have influenced the work reported in this article. The authors are inventors on a patent based on ScpA owned by the University of Limerick, which is licensed to a commercial entity.

## Data Availability

7yzx: ScpA from Streptococcus pyogenes, D783A mutant (Original Data) (XRDa). ScpA from Streptococcus pyogenes, D783A mutant (Original Data) (wwPDB).
